# The Value of Mechanistic Experiments to Target the Shared Neural Circuitry of Parenting and Addiction: The Potential for Video Feedback Interventions

**DOI:** 10.3389/fpsyg.2021.703948

**Published:** 2021-10-04

**Authors:** Ann-Marie Y. Barrett, Kavya R. Mudiam, Philip A. Fisher

**Affiliations:** Department of Psychology, University of Oregon, Eugene, OR, United States

**Keywords:** parenting, addiction, translational neuroscience, intervention, mechanisms of change

## Abstract

Certain neural processes that underlie addiction are also central to parenting, notably stress and reward. Parenting interventions that incorporate the unique context of caregivers with addiction have demonstrated some success: However, real-world implementation of evidence-based interventions can be difficult with this population. Video feedback interventions are an especially promising approach to reach parents who experience barriers to participation, particularly caregivers with addiction. A translational neuroscientific approach to elucidating the mechanisms of change in these interventions will aid the delivery and success of this method and advance theory surrounding parenting in the context of addiction. Along these lines, we provide an example of one video feedback intervention, Filming Interactions to Nurture Development, that will serve as such a mechanistic experiment.

## Introduction

Caregivers of young children constitute a notable proportion of the population of individuals with substance use disorders. Increasing rates of substance use, particularly opioid use, in this subgroup reflect an already-emergent public health concern (Terplan, 2020; Goetz et al., 2021; National Institute on Drug Abuse, 2021) that has been amplified by the impact of the COVID-19 pandemic (Mota, 2020; Ornell et al., 2020; Rogers et al., 2020; Sun et al., 2020). The negative intergenerational consequences of substance use problems are well documented, reflected by an increased vulnerability to addiction and psychopathology (Merikangas et al., 1998; Clark et al., 2004; Knight et al., 2014). Concomitant consequences for offspring of caregivers with substance use disorders are evident throughout development; during childhood, this may include difficulties with temperament, attachment, aggression, cognition, and speech and language (Shulman et al., 2000; Barnard and McKeganey, 2004).

The significance of these consequences has fueled research examining the intersectionality of substance use and parenting (Niccols et al., 2012; Moreland and McRae-Clark, 2018). Parental involvement is hypothesized to be the primary route through which addiction impacts parenting and subsequent parent and child wellbeing (Suchman and Luthar, 2000). Intrusive or disengaged parenting styles often co-occur with substance use problems (Burns et al., 1997; Hans et al., 1999; Jacques et al., 2020), and the extremes of these styles result in child maltreatment which is associated with caregiver substance use (Freisthler and Kepple, 2019). Recently, a greater focus on how parenting might impact addiction suggests that parenting itself confers a unique form of stress that may increase risk of substance use (Rutherford and Mayes, 2019).

Understanding the mechanisms underlying the interaction between parenting and addiction provides useful knowledge for identifying intervention targets that promote healthy parent and child outcomes. Although those mechanisms can be studied at multiple levels (e.g., cognitive or behavioral), investigating the neurobiological correlates of parenting processes that mediate responsive caregiving in the context of addiction draws upon the wealth of knowledge provided in these separate literatures (i.e., neurobiology of parenting and neurobiology of addiction; Rutherford et al., 2020). Notably, and with some important exceptions that we describe below, there is limited extant research in this area.

In this paper, we provide rationale for applying a translational neuroscientific approach to intervention research aimed at helping parents struggling with addiction. Translational neuroscience necessitates a conceptual model of disorder that identifies specific processes supported by neurobiological systems with respect to any relevant moderators (Fisher and Berkman, 2015). Interventions can engage these systems to promote desired outcomes. This approach has the potential to increase the specificity or direction of proposed intervention targets, elucidate individual differences in intervention response at the neurobiological level, and lead to the application of precision interventions based on biobehavioral markers. Parents with addiction remain a particularly difficult population to engage in parenting interventions. Our goal is to draw upon the growing knowledge of the shared neural circuitry of parenting and addiction to advance these efforts. To explicate our perspective, we discuss a neurobiological mechanistic experiment with the potential to address barriers to engagement with opioid-using mothers.

### Neural Intersection of Parenting and Addiction

Certain cognitive and affective processes, such as those related to stress and reward, are central in both addiction and parenting. For example, in the context of addiction, non-medically used psychoactive substances (hereafter referred to as drugs) elicit reward responses, and stress often precedes subsequent use (Sinha et al., 2005). In the context of parenting, children elicit reward responses in caregivers (Ferrey et al., 2016), and parenting stress influences family interactions and function (Deater-Deckard, 1998).

Activation of stress and reward neural circuitry across these contexts plausibly induces a mutually informed interaction wherein system responses in one context impact the response in another (Rutherford et al., 2011). Although the overlapping neurobiology of these two contexts has not yet been widely studied, researchers have investigated neural changes in these contexts separately. During the development of drug dependence, neural reward systems are highly activated in response to drug use, and this positive reinforcement maintains drug-seeking behavior (Koob and Volkow, 2010). As addiction becomes reinforced primarily through withdrawal and anticipation, stress-related neural systems generate negative reinforcement when a substance provides relief.

The dysregulation of these stress and reward systems associated with addiction must be considered alongside the neuroendocrine changes elicited by the onset of parenthood. Research on the maternal brain suggests that rising levels of hormones (e.g., oxytocin and cortisol) correspond with stress and reward circuit activation central to sensitive caregiving (Atzil et al., 2011; Swain et al., 2019). This circuitry, which includes subcortical (amygdala, insula, and ventral striatum) and cortical (anterior cingulate cortex, prefrontal cortex, and precuneus) regions, supports emotion regulation and executive function (Swain and Ho, 2017). Many of these regions overlap with those impaired in addiction, indicating that regional disruption can echo throughout connected circuits (Rutherford et al., 2011).

In addition to the impact of general addiction processes, the type of drug used can impart unique physiological changes. While there is little research on the effect of extended opioid use on parental brain circuitry, the role of endogenous opioids in maternal stress and emotion regulation and reward processing highlights the importance of investigating that effect (Benarroch, 2012; Swain et al., 2019).

**Figure 1:**
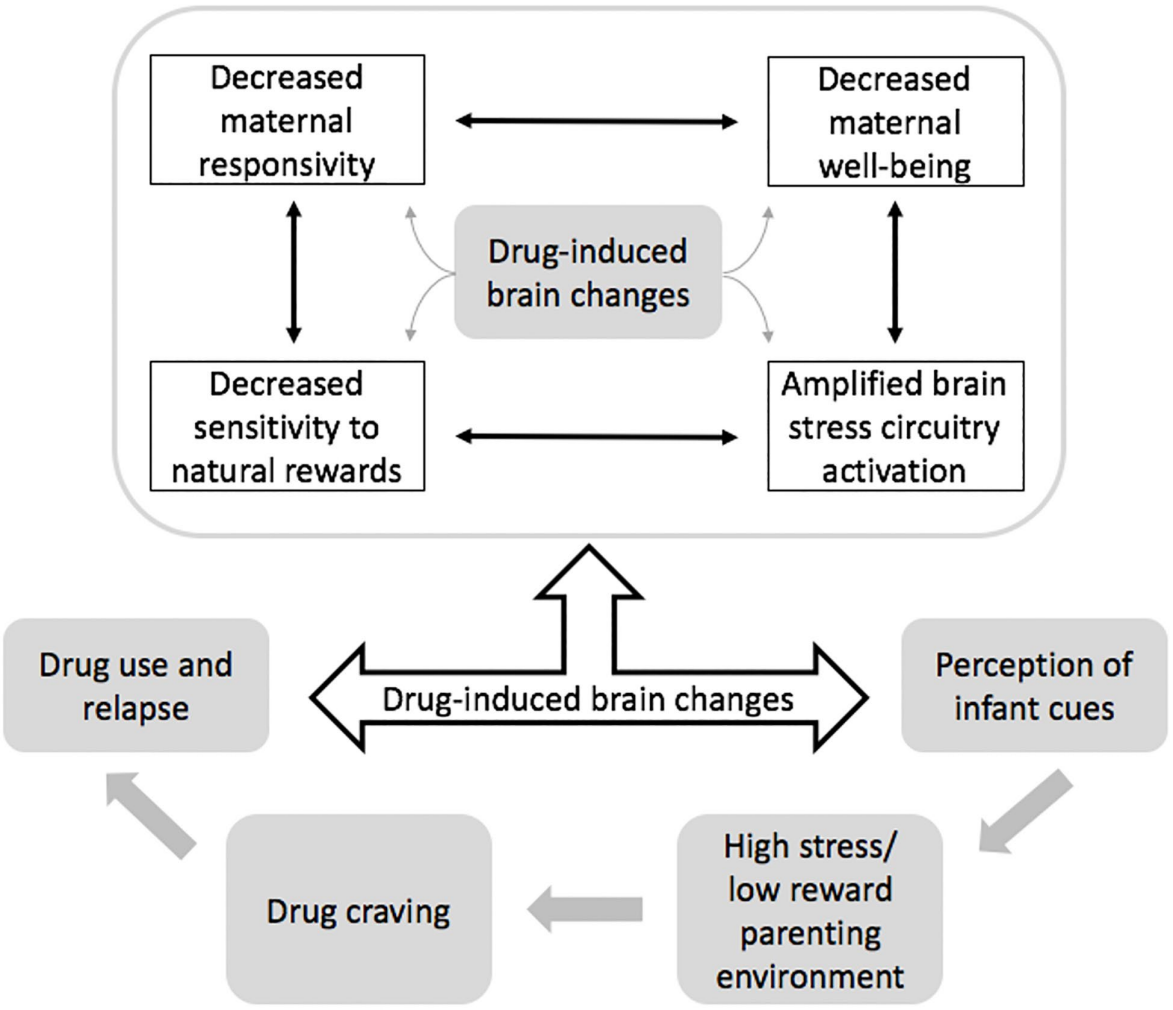
Role of brain changes in the relationship between drug use and parenting (adapted from Rutherford et al., 2011). Drug use causes many brain changes (gray), which can influence each other (black) by amplifying/diminishing alterations depending on context.

As shown in Figure 1 (adapted from Rutherford et al., 2011), we highlight a “reciprocal influence model” characterizing the bidirectional effects of parenting and addiction. Reward system dysregulation may decrease saliency of social or relational rewards that are pervasive in parenting and underlie secure parent-infant attachment. Additionally, stress and emotion dysregulation in caregiving roles could intensify craving and drug-seeking behaviors. Parents with addiction might find caring for infants less rewarding and more stressful than parents without addiction, creating a cycle that maintains substance use.

A reward-stress dysregulation model of addiction and parenting proposed by Rutherford and Mayes (2017) incorporates these dynamic neural interactions. Implicated brain regions include the prefrontal cortex, ventral tegmental area, and nucleus accumbens within reward circuitry and the hypothalamic-pituitary-adrenal axis and extended amygdala within stress circuitry (Rutherford et al., 2011). Studies examining parental reactions to infant cues provide evidence for this model. Altered neural activity in response to infant stimuli suggests that positively valenced infant cues may be less salient and negatively valenced cues may be more stressful for parents with addiction, corroborating observations of parental disengagement (Landi et al., 2011; Kim et al., 2017; Rutherford et al., 2017, 2020).

### Interventions at the Intersection of Responsive Caregiving and Addiction Treatment

Instances of household instability and child neglect along with co-occurring mental health difficulties underscore the need for effective interventions for parents with addiction (Barnard and McKeganey, 2004; Barlow et al., 2019). Despite the significant social costs of this problem, complications to intervening within this population persist (Daley, 2013). These stem from additional comorbidities and social problems including time constraints, affordability, transportation difficulties, mistrust in clinicians, fear of losing children, and shame (Acevedo et al., 2012; Guerrero et al., 2015; Matsuzaka and Knapp, 2020).

Although dual treatment for substance use and parenting results in improvements in both domains (Neger and Prinz, 2015), directing substance-using caregivers to optimal interventions remains difficult. Different interventions target multiple and varying mechanisms of change, and effectiveness may depend on parenting stage or the substance of abuse (Neger and Prinz, 2015; Cioffi et al., 2019). Rigorous randomized clinical trials can help determine the influence of specific hypothesized mechanisms on outcomes, thereby establishing causal evidence which allows the identification of effective strategies to improve outcomes and advances theory.

Many interventions for parents with substance use disorders are based on attachment and relational theories, integrating varying aspects of caregiver responsivity, mentalization, emotion and stress regulation, and mindfulness to improve parent and child outcomes. Examples include Relational Psychotherapy Mothers’ Group (Luthar and Suchman, 2000), Practicing Safety Mindfulness Project for Mothers in Drug Treatment (Short et al., 2017), Mothers and Toddler Program (MTP; Suchman et al., 2010), Parenting under Pressure (PuP; Barlow et al., 2013), Attachment and Biobehavioral Catch-Up (ABC; Berlin et al., 2014), and a modified ABC (mABC) specifically for mothers using opioids (Labella et al., 2021). One promising component of many of these interventions is the inclusion of video feedback, where clinicians or instructors provide mothers feedback about their interactions with their child, based on recorded interactions.

#### Video Feedback Design

Video feedback has been employed in parenting interventions where caregiver-child interactions are filmed. Videos are useful for capturing the reciprocal influence parents and children exert on each other. Trained therapists use recordings to replay and personalize feedback to parents. This method allows researchers to highlight parenting skills in a naturalistic environment, often in participants’ homes. Although the specificity of this intervention approach varies, many video feedback interventions aim to encourage supportive interactions between parents and their children. A review of 29 experimental studies revealed that video feedback interventions successfully resulted in a change of maternal sensitivity and more positive parent and child behaviors (Balldin et al., 2018).

Given the disruption of maternal sensitivity in many parents with addiction, this style of intervention might be especially well suited for substance-using parents. However, the true measure of intervention effectiveness does not end within a research context but extends to the feasibility of disseminating and evaluating that intervention in community settings for those who could benefit most. Barriers to accessing treatment are a primary concern for parents with addiction and often have roots in systemic inequalities and racism prevalent in society (Acevedo et al., 2012; Guerrero et al., 2015; Matsuzaka and Knapp, 2020). The natural environment context of these parenting interventions provides an avenue to partially address inequity by increasing availability to caregivers. Furthermore, the salient personalized stimuli present in video feedback could both increase participant interest and facilitate the transition from in-session learning to home integration. Still, the relative utility of video feedback in comparison with other interventions in real-world settings remains an open subject that necessitates the scrutiny of future research.

Research on video feedback interventions containing proposed mechanisms of change helps discern employable components to further explore and integrate into interventions for caregivers with substance use disorders. Although there is overlap among hypothesized mechanisms, distinct interventions have not targeted mechanisms uniformly. A cursory comparison of different video feedback interventions illustrates this. PuP uses psychoeducation and mindfulness skill building to target the proposed mechanisms of change: parental emotion regulation, representation quality, and mentalization about own and child’s emotions (Dawe and Harnett, 2007; Barlow et al., 2019). Similarly, MTP hypothesizes that changes in maternal mentalization, representations of one’s child, and the therapeutic alliance lead to positive outcomes (Suchman et al., 2010, 2011, 2012). Mechanisms in ABC and mABC include changing nurturing behaviors during child distress, improving synchronous interactions, and reducing frightening behavior (Dozier and Bernard, 2017).

These video interventions have yielded improvements in parenting and substance use behaviors (Dawe and Harnett, 2007; Suchman et al., 2011, 2012; Barlow et al., 2013, 2019; Dozier and Bernard, 2017). However, targeting multiple mechanisms makes it difficult to identify which are most effective and for whom. Furthermore, these interventions have been predominantly tested among pregnant and postnatal mothers, limiting generalizability across other caregivers. There may be a significant benefit to interventions founded on a specific process of change and inclusive of a range of caregivers. To illustrate these considerations, we describe the Filming Interactions to Nurture Development (FIND) intervention.

##### Filming Interactions to Nurture Development

Filming Interactions to Nurture Development is a strength-based video feedback intervention with a clearly proposed conceptual model, protocol, and potential to achieve notable impact at scale. FIND was designed to primarily target responsive caregiving and consequently improve caregiver and child outcomes. This design is informed by research on serve and return interactions that are critical to healthy development, a process where caregivers provide contingent, supportive responses to child-initiated cues (Dozier et al., 2002; Fisher et al., 2006; Shonkoff and Bales, 2011). Almost all parents—even those at highest risk—engage in responsive caregiving to some extent. Thus, the goal of FIND is not to teach responsive caregiving but to highlight the occurrence of caregivers’ own responsiveness with video clips.

Across 10 sessions with a family (five filming sessions and five coaching sessions), coaches share video compilations of positive micro-social interactions between parent and child to encourage parents to identify and increase the frequency of specific serve and return components (e.g., Sharing the Child’s Focus, Supporting and Encouraging, Naming, Back and Forth, and Ending and Beginnings). Further details of the program can be found in Fisher et al. (2016).

Emerging evidence suggests that FIND is particularly effective for high-adversity families. A preliminary study with low-income fathers provides support for FIND’s conceptual model that caregiver and child improvements occur through increases in responsive parenting (Schindler et al., 2017). Fathers with high levels of childhood adversity also experienced an increase in parental self-concept and a decrease in their child’s behavioral problems. Another preliminary study suggests that FIND participation alters brain functioning in regions related to inhibitory control for low-income mothers, which is noteworthy given the relatively small intervention dose (Giuliani et al., 2019).

## Current Perspective

**Figure 2:**
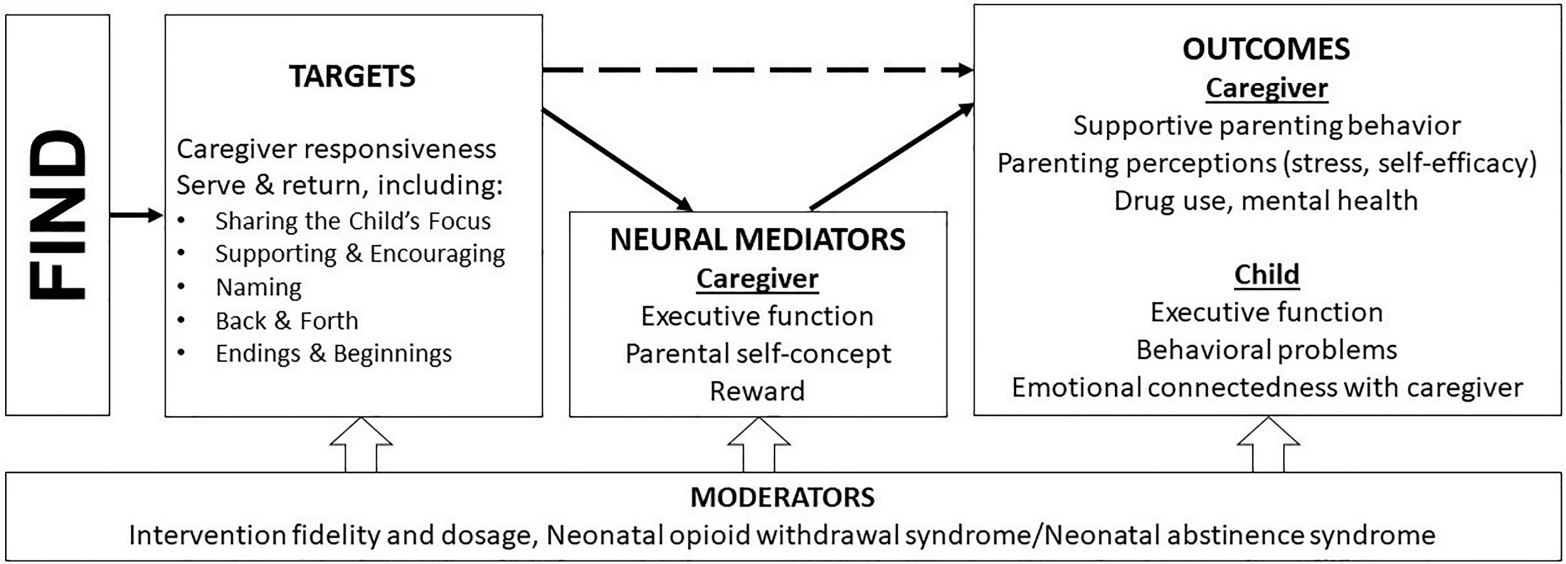
FIND conceptual model.

Parents with opioid addiction tend to experience difficulty understanding and reacting to child cues, exhibiting greater irritability and decreased responsiveness compared to other parents (Romanowicz et al., 2019). Gaps in the literature highlight the need to identify whether parenting interventions engage the neural circuitry that is implicated in such parenting difficulties and influenced by addiction. The conceptual model of FIND (see Figure 2) proposes a testable mechanism through which FIND might serve mothers recovering from opioid misuse. In recognition of this shared neural circuitry, future research on FIND will test mediating roles of brain changes related to parental self-concept, executive function, and reward. Following principles of translational neuroscience, this model targets the drug-induced brain changes that impact maternal responsivity and wellbeing identified by Rutherford et al. (2011). This line of research not only identifies process-level mechanisms, but also may elucidate why intervention effects might persist in some caregivers but not others.

Currently, a randomized clinical trial of FIND is being conducted with 200 mothers in opioid use treatment or recovery with children aged 0–36 months. Half the parents are assigned to a control condition, which includes alternating child development information sessions and supportive observations of caregiver-child interaction. This study will both evaluate the effect of FIND on responsive caregiving and test whether observed changes in parenting and addiction circuitry, which have considerable overlap (Rutherford et al., 2011), accompany behavioral improvements. Regions that underlie parental self-concept (e.g., medial prefrontal cortex) and inhibitory control (e.g., insula and inferior frontal gyrus) will be examined before and after intervention. Tasks will also be employed that allow for the disaggregation and assessment of motivation and reward, known to be affected by drug addiction (Kelley and Berridge, 2002), at behavioral and neural levels. This will illuminate whether specific phenotypes exist in mothers with opioid use issues (e.g., low motivation-high reward; high motivation-low reward), whether these phenotypes respond differently to the intervention, and how sensitive these processes are to change in this context. We expect that, across all neurocognitive functions under investigation, intervention effects may be moderated by intervention delivery variables (e.g., fidelity and dosage) and infant opioid exposure/withdrawal.

Investigating the neurocognitive mediators of responsive caregiving improvements and subsequent outcomes allows for more informed intervention alteration and adaptation in real-world contexts. Many evidence-based interventions fail to achieve impact when delivered at scale, and others only yield modest effects and fail to support families at highest risk (Shonkoff, 2010). For substance-using parents of young children, the effectiveness of the intervention in real-world community settings is of heightened concern.

The design process employed in developing FIND was intended to proactively tackle large-scale dissemination concerns of both scalability and real-world efficacy for high-risk populations. FIND’s descriptive, as opposed to analytical, coaching delivery permits more people (rather than only those with specialty knowledge or degrees) to implement the program, enhancing scalability. Parents of addiction may respond particularly well to the specific, strength-based nature of the program which directly addresses non-drug reward hypo-responsivity by increasing the inherent rewards of parenting without imposing feelings of shame or guilt that might accompany skill-learning present in other interventions. Previous research suggests that participation increases responsive caregiving at lower doses than many existing interventions (Schindler et al., 2017), possibly due to the exclusive practice of showing caregivers positive instances of their own responsive caregiving. This avoids the trap of including too many untested components within a single intervention, enhancing efficacy. Given these implementation considerations and preliminary findings, evidence suggests that FIND may be especially effective for caregivers who are traditionally difficult to reach.

## Discussion

The need for effective resources and interventions for caregivers with addiction necessitates carefully planned research that acknowledges their shared and unique contexts. The reciprocal influence model posits that drug-induced brain changes are implicated in a cluster of cognitive, behavioral, and affective caregiver changes that directly impact child interactions and consequently create a high-stress parenting environment that increases risk for further drug use. Such models, informed by the neurobiology of shared processes, have potential to be more efficient and scalable than those without a clearly proposed and tested mechanism.

Building, testing, and disseminating effective interventions for this population are complicated by ongoing challenges. Individuals come in with varying skills and may be experiencing concurrent and related stress or adversity. One intervention cannot be made to suit all caregivers and some caregivers may need more or less support. Continued research that connects the growing knowledge of neurobiology related to caregiving and substance use with mechanistic intervention evaluation will allow scientists to investigate what works, why, and for whom.

## Data Availability Statement

The original contributions presented in the study are included in the article/supplementary material, and further inquiries can be directed to the corresponding author.

## Author Contributions

PF and A-MB conceived the idea. A-MB and KM wrote the manuscript. All authors contributed to the final version of the manuscript.

## Funding

The authors gratefully acknowledge grant P50 DA048756 from the National Institute on Drug Abuse to PF.

## Conflict of Interest

The authors declare that the research was conducted in the absence of any commercial or financial relationships that could be construed as a potential conflict of interest.

## Publisher’s Note

All claims expressed in this article are solely those of the authors and do not necessarily represent those of their affiliated organizations, or those of the publisher, the editors and the reviewers. Any product that may be evaluated in this article, or claim that may be made by its manufacturer, is not guaranteed or endorsed by the publisher.
